# Inhibition of MAOB Ameliorated High-Fat-Diet-Induced Atherosclerosis by Inhibiting Endothelial Dysfunction and Modulating Gut Microbiota

**DOI:** 10.3390/nu15112542

**Published:** 2023-05-30

**Authors:** Zhen Tian, Xinyue Wang, Tianshu Han, Maoqing Wang, Hua Ning, Changhao Sun

**Affiliations:** National Key Discipline Laboratory, Department of Nutrition and Food Hygiene, School of Public Health, Harbin Medical University, Harbin 150086, Chinawangxinyue966@163.com (X.W.); snowcalendar@126.com (T.H.); wang_maoqing@126.com (M.W.); ninghua188@126.com (H.N.)

**Keywords:** monoamine oxidase B, oxidative stress, endothelial dysfunction, microRNA, gut microbiota

## Abstract

In this study, monoamine oxidase B (MAOB) was activated under pathological conditions, and was the novel source of cardiovascular reactive oxygen species (ROS). ROS-induced endothelial dysfunction results in sustained and chronic vascular inflammation, which is central to atherosclerotic diseases. However, whether MAOB regulates endothelial oxidative stress and its related mechanism and whether gut microbiota mediates the anti-atherosclerosis effect of MAOB inhibitor remains unclear. In our study, MAOB expressions were elevated in high-fat diet (HFD) fed mice aortas, but only in vascular endothelial cells (not in smooth muscle cells). MAOB small interfering RNA significantly attenuated the palmitic-acid (PA)-induced endothelial oxidative stress and dysfunction. Furthermore, RNA-sequencing data revealed that MAOB knockdown decreased the levels of proinflammatory and apoptotic gene induced by PA. Microarray analysis and qPCR assay showed that miR-3620-5p was significantly decreased under the HFD condition. The dual-luciferase reporter, Western blot and qPCR assay confirmed that miR-3620-5p directly regulated MAOB by binding to its mRNA 3′UTR. Moreover, inhibition of MAOB by selegiline significantly ameliorated endothelial dysfunction and reduced atherosclerotic burden in HFD-fed *ApoE^−/−^* mice. Finally, 16S rRNA sequencing showed that selegiline significantly altered the community compositional structure of gut microbiota. Specifically, selegiline treatment enriched the abundance of *Faecalibaculum* and *Akkermansia*, decreased the abundance of unclassified_f__Lachnospiraceae, Desulfovibrio, and Blautia, and these genera were significantly correlated with the serum biochemical indices. Taken together, our findings showed that MAOB controlled endothelial oxidative stress homeostasis, and revealed the anti-atherosclerotic effect of selegiline by ameliorating endothelial dysfunction and modulating the composition and function of gut microbiota.

## 1. Introduction

Atherosclerosis is the primary cause of atherosclerotic cardiovascular diseases (ACVDs) such as myocardial infarction, stroke, and peripheral artery disease [1]. Atherosclerosis is a chronic lipid-driven progressive inflammatory disease, characterized by excessive cholesterol deposition and fibrous lesions in the atrial wall [2,3]. Endothelial dysfunction represents the earliest detectable changes in the life history of an atherosclerotic lesion, which is an independent risk factor for ACVDs [4,5,6]. Given its involvement in all stages of ACVDs progression and its predictive significance for cardiovascular events [5], there has been considerable interest in exploring the underlying mechanisms of endothelial dysfunction and seeking effective therapeutic targets for atherosclerosis based on mechanism-guided discovery.

Oxidative stress plays a key role in endothelial dysfunction and atherogenesis. All established risk factors for atherosclerosis enhance oxidative stress and induce endothelial nitric oxide synthase (eNOS) uncoupling in the vascular wall [7,8]. Uncoupling of eNOS not only reduces endothelial NO production, but also enhances oxidative stress [9]. Reactive oxygen species (ROS) plays a pivotal role in mediating the production and secretion of adhesion molecules [10,11], thus linking oxidative stress with inflammation and endothelial dysfunction [12]. Increased evidence shows that monoamine oxidase (MAO) is activated under pathological conditions and is the novel source of cardiovascular oxidative stress [13,14].

In mammals, MAOs are flavin enzymes located in the outer mitochondrial membrane, that catalyze oxidative deamination of amine neurotransmitters and vasoactive amines to generate hydrogen peroxide (H_2_O_2_) [14]. Two isoforms of MAO (MAOA and MAOB) have been identified, but they differ in substrate specificity, inhibitor affinity, relative expression, and tissue localization [15]. Recently, it has been demonstrated that MAOB was dominantly activated while MAOA was relatively mild in the aorta of *ApoE^−/−^* mice fed with a high-cholesterol diet, and inhibition of MAOB, but not MAOA, reduced the atheroma area [16]. Other important findings showed that MAOB-related ROS production promotes a pro-oxidative status of endothelial dysfunction in coronary heart disease patients, and the MAOB inhibitor selegiline may be useful in restoring the endothelial response [13]. These previous findings supported the notion that MAOB may play a pivotal role in controlling endothelial oxidative stress and participate in the pathogenesis of ACVDs. However, the previous studies have not focused on the exact mechanism by which MAOB regulates endothelial oxidative stress and the upstream regulator of elevated MAOB, and explored whether gut microbiota mediates the therapeutic effects of selegiline on atherosclerosis.

In this study, we discovered that endothelial MAOB was activated under high-fat diet (HFD) exposure. To clarify the precise molecular pathway regulated by MAOB in endothelial cells under high-fat stress, we performed RNA-sequencing (RNA-seq) on palmitic-acid (PA)-treated human umbilic vein endothelial cells (HUVECs) with MAOB knockdown. miRNA microarray analysis was used to investigate the specific miRNA regulating MAOB expression. Moreover, we also investigated the regulatory effect of selegiline on gut microbiota in *ApoE^−/−^* mice fed with HFD to alleviate atherosclerosis, and provided new insights into developing selegiline as a promising treatment strategy for ACVDs.

## 2. Materials and Methods

### 2.1. Animals

Male C57BL/6 and *ApoE^−/−^* mice (used to construct atherosclerosis models) aged 7–8 weeks were purchased from Vital River Laboratory Animal Technology Co. (Beijing, China). The mice were housed individually with controlled temperature at 21 ± 2 °C and a 12 h light/dark cycle under pathogen-free conditions, with food and water ad libitum. C57BL/6 mice were randomly supplied with normal diet (ND, AIN-93M, 10% kcal from fat) or HFD (AIN-93M, 45% kcal from fat) for 16 weeks. Selegiline is an irreversible inhibitor of MAOB, which is a main pathway of catecholamine degradation in the brain, and an important source of ROS in the vascular system. The *ApoE^−/−^* mice were randomly divided into four groups: ND plus PBS, ND plus selegiline, HFD plus PBS and HFD plus selegiline. Mice received selegiline (MedChemExpress, Shanghai, China) at a dose of 0.6 mg/kg body weight every other day for 6 weeks by intraperitoneal injection (i.p.) after a 10-week exposure to ND or HFD feeding. The dose of selegiline was selected based upon one previous report [17], and the calculation from a simple practice guide for dose conversion between mice and human [18]. The oral dose of selegiline used in the human therapy is 5–10 mg/day [19]. In terms of dose per unit of body weight, the equivalent dose for mice is 9 times that for humans (0.6–1.3 mg/kg/day) based on a guide for dose conversion between mice and humans. The dose conversion ratio between oral and i.p. is 1:0.3. At the end of the experiments, the mice were sacrificed with pentobarbital administration (100 mg/kg, i.p., once, Sigma-Aldrich, St. Louis, MI, USA), and blood and tissues were collected.

All the experimental procedures involving animals were performed according to the guidelines of the experimental use of laboratory animals of Harbin Medical University and approved by the Harbin Medical University Institutional Animal Care and Use Committee (Approval Code: HMUIRB2022004PRE).

### 2.2. Cell Culture and Treatment

HUVECs and human aortic endothelial cells (HAECs) purchased from ATCC (USA) were cultured in a DMEM medium (Thermo Fisher Scientific, Waltham, MA, USA) containing 10% fetal bovine serum (Thermo Fisher Scientific, Waltham, MA, USA). Human smooth muscle cells (HSMCs) were maintained in a DMEM/F12 medium supplemented with a 10% fetal bovine serum. The culture was maintained at 37 °C in a humidified 5% CO_2_ incubator. HUVECs, HAECs and HSMCs were treated with PA (Sigma, St. Louis, MI, USA) at different doses for 24 h, and then used for the indicated assays. Cells were transfected with 50 nmol/L miR-3620-5p mimics or negative controls (miR-NC) (GenePharma, China) using the Lipofectamine 2000 reagent (Thermo Fisher Scientific, Waltham, MA, USA) for 24 h according to the manufacturer’s protocol. HUVECs were transfected with 100 nmol/L MAOB siRNA (si-MAOB) or negative control siRNA (si-NC) (Dharmacon, Lafayette, CO, USA) for 24 h, then treated with or without PA (500 μmol/L) for 24 h. The sequences of the MAOB siRNA and the si-NC were GGCGAGAGAUUCCGAGUGA and UGGUUUACAUGUCGACUAA, respectively. After reaching an 80% confluence, the cells were incubated with 500 μmol/L PA for 24 h to establish a hyperlipidemia cell model and then treated with or without different concentrations of selegiline (2 μmol/L and 10 μmol/L) for 24 h.

### 2.3. Serum Biochemical Analysis

Serum samples were separated from nonheparinized blood when the mice were euthanized and assayed for triglyceride (TG), total cholesterol (TC), low-density lipoprotein cholesterol (LDL-C), glucose, high-density lipoprotein cholesterol (HDL-C), alanine transaminase (ALT), and aspartate transaminase (AST) with the automatic blood analyzer (Hitachi 7100, Tokyo, Japan) using standard enzymatic colorimetric techniques.

### 2.4. Immunohistology of Atherosclerotic Lesions

The aortic roots along with the basal portion of the heart were fixed with a 4% paraformaldehyde, followed by embedding in an OCT compound (SAKURA, Torrance, CA, USA). To assess the atherosclerotic lesion in aortic roots, serial cross-sections (7 μm thick) of aortic roots were prepared from the site where the three aortic valves appeared. The frozen sections were then stained with hematoxylin and eosin (H&E) (Solarbio, Beijing, China). Immunohistochemistry was performed on the aortic root for the expression of IL-6. Briefly, 7 μm thick sections were stained with primary antibody overnight at 4 °C, then the secondary antibody conjugated with horseradish peroxidase was added. The DAB substrate solution was used to induce the formation of colored precipitate at the tissue antigen-binding sites, and then the hematoxylin was added.

### 2.5. Oxidative Stress Detection

The production of intracellular superoxide was measured using the Reactive Oxygen Species Assay Kit (Beyotime, Shanghai, China), and the MDA levels in HUVECs were determined by the MDA assay kit (Nanjing Jiancheng Co., Nanjing, China) according to the instructions of the manufacturer. Briefly, HUVECs cultured in 6-well plates were subjected to MAOB siRNA infection or different concentrations of selegiline treatment followed by PA (500 μmol/L) treated for 24 h; then, the cells were incubated with DCFH-DA (10 μmol/L) at 37 °C for 20 min. The fluorescent images were captured by fluorescent microscope. Malondialdehyde (MDA) is the final product of lipid peroxidation. For MDA determination, after being subjected to different treatments, the HUVECs were washed with PBS and scraped into solution. Then, the MDA concentrations were detected according to the instructions. MDA concentrations were normalized by total protein contents of cell lysate. The sections of aortic tissue were stained with dihydroethidium (DHE) for 20 min. In addition, the slides were counterstained with DAPI to stain the cell nucleus. Images were obtained using a Nikon ECLIPSE Ti confocal laser-scanning microscope (Nikon, Tokyo, Japan).

### 2.6. RNA Isolation and Quantitative Real Time PCR (qPCR)

Total RNAs from tissues and cells were extracted using a TRIzol reagent (Invitrogen, Waltham, MA, USA). cDNA synthesis was performed using the High-Capacity cDNA Reverse Transcription Kit (Applied Biosystems) according to the manufacturer’s instructions. The SYBR Green PCR Kit (Applied Biosystems) was used to quantify the relative RNA levels of miR-3620-5p, MAOB, IL-1α, IL-1β and IL-6. Relative expression was normalized to that of the reference gene (U6 for miRNA; β-actin for mRNA) and calculated using the 2^−ΔΔCt^ method. Additionally, all primers were purchased from Sangon (Shanghai, China), and the sequences of primers are listed in Table 1.

### 2.7. Western Blot Analysis

Western blot analysis was carried out to measure the expression levels of MAOB, VCAM-1, p-eNOS, eNOS, and β-actin proteins. In brief, protein extracts were prepared from cells or tissues in an RIPA buffer supplemented with protease inhibitor and phosphatase inhibitor. The concentrations of protein were quantified by a BCA kit (Solarbio, Beijing, China). Approximately 50 μg of a protein sample was subjected to SDS-PAGE separation, transferred to a PVDF membrane (Millipore Corp, Burlington, MA, USA), and then incubated with a primary antibody (anti-MAOB antibody (Abcam, Cambridge, UK, 1:1000), an anti-VCAM-1 antibody (Bioster, Taiwan, China, 1:1000), an anti-p-eNOS antibody (Cell Signaling Technology, Danvers, MA, USA, 1:1000), an anti-eNOS antibody (Cell Signaling Technology, Danvers, MA, USA, 1:1000) and an anti-β-actin antibody (Cell Signaling Technology, Danvers, MA, USA, 1:1000) at 4 °C overnight. After incubation with AP-conjugated secondary antibody for 1 h, the blots were developed with the alkaline phosphatase detection system. The immunoreactive bands were imaged by the FluorChemE system (Santa Clara, CA, USA). Densitometric quantification of band intensity was carried out with the ImageJ software, and the relative expression level of the target protein was normalized to β-actin.

### 2.8. Luciferase Activity Assay

HEK293 cells were seeded in 24-well plates. For testing the MAOB 3′UTR and miR-3620-5p interaction, either a wild-type (WT) or a mutation (MUT) of MAOB 3′UTR fragment was inserted into pGLO vector (Genecreate, Wuhan, China). A total of 100 ng plasmid of 3′UTR-WT and 3′UTR-MUT, and 50 nmol miR-3620-5p mimics or miR-NC were co-transfected into HEK293 cells using Lipofectamine 2000. After transfection for 48 h, luciferase activities were detected by the Dual-Luciferase Reporter Assay System (Promega, Madison, WI, USA).

### 2.9. RNA-Seq Analysis

Total RNA was isolated using the TRIzol reagent, and the integrity of the RNA was assessed with the Agilent 2100 bioanalyzer. For RNA-seq, complementary DNA libraries were created using the NEBNext^®^ Ultra™ RNA Library Prep Kit for Illumina according to the manufacturer’s instructions. To ensure the quality and reliability of data analysis, raw data filtered out reads with an adapter or with low sequencing quality. The clean reads were mapped to the reference genomes with HISAT2 software to obtain the location information of reads. Then, samtools was used to convert the files obtained in the above steps into a binary bam format that could store the comparison information. Next, the fragments per kilobase of exon per million mapped fragments value for each identified gene was calculated using the featureCounts tool of the subread software. Differentially expressed genes (DEGs) were identified with DESeq2 according to the following two criteria: (1) a fold change > 2 and (2) a corrected *p* value < 0.05. Finally, the ggplot2 package was used to create a volcano plot showing the fold changes and *p* values of all genes.

### 2.10. Apoptotic Assay

Cells were stained with an Annexin V-FITC/PI Apoptosis Detection Kit according to the manufacturer’s instructions (Beyotime, Shanghai, China) and analyzed by a BD LSRFortessa Cell Analyzer (Becton Dickinson, Franklin Lakes, NJ, USA).

### 2.11. 16. S ribosomal RNA (rRNA) Sequencing and Bioinformatics Analysis

Total genomic DNA of fecal samples was extracted using the Omega Stool DNA Kit (Omega Bio-Tek, Norcross, GA, USA) following the manufacturer’s instructions, and the DNA extraction quality was evaluated using a 1% agarose gel. The V3–V4 region of the bacterial 16S rRNA gene was amplified using the primers 338F (ACTCCTACGGGAGGCAGCAG) and 806R (GGACTACHVGGGTWTCTAAT). The amplification procedure consisted of an initial denaturation at 95 °C for 5 min, 30 cycles of denaturation at 95 °C for 45 s, annealing at 50 °C for 30 s, and elongation at 72 °C for 30 s, final extension at 72 °C for 5 min, and storage at 4 °C. The PCR products were purified using a Qiagen Gel Extraction Kit (Qiagen, Hilden, Germany). Subsequently, paired-end sequencing was performed using an Illumina MiSeq PE300 platform (Illumina, San Diego, CA, USA).

Alpha diversity is applied in analyzing the complexity of species diversity for a sample through 5 indices, including Chao, ACE, Sobs, Shannon, and Pd. Principal component analysis (PCA) and Principal Coordinate Analysis (PCoA) were performed to obtain principal coordinates and visualize from complex, multidimensional data. Community Bar diagram showed the composition of the microbiome in the samples. Linear discriminant analysis effect size (LEfSe) was performed to identify the features most likely to explain the differences between different groups. Spearman correlation analysis was performed to reveal the relationship between different microbial taxa and host parameters.

### 2.12. Statistical Analysis

All data in this study were presented as mean ± SEM. The significance of the difference between two groups was determined by using unpaired two-tailed Student *t*-test. One-way ANOVA was used to assess differences between multiple groups followed by a post hoc test to assess statistical significance. Biological experimental replicates in each group are shown in the figures. *p* < 0.05 was considered statistically significant. The statistical analysis was performed by GraphPad Prism 9.0 or SPSS 20.0 software.

## 3. Results

### 3.1. MAOB Expression Was Activated in HFD-Exposed Vascular Endothelial Cells

**Figure 1:**
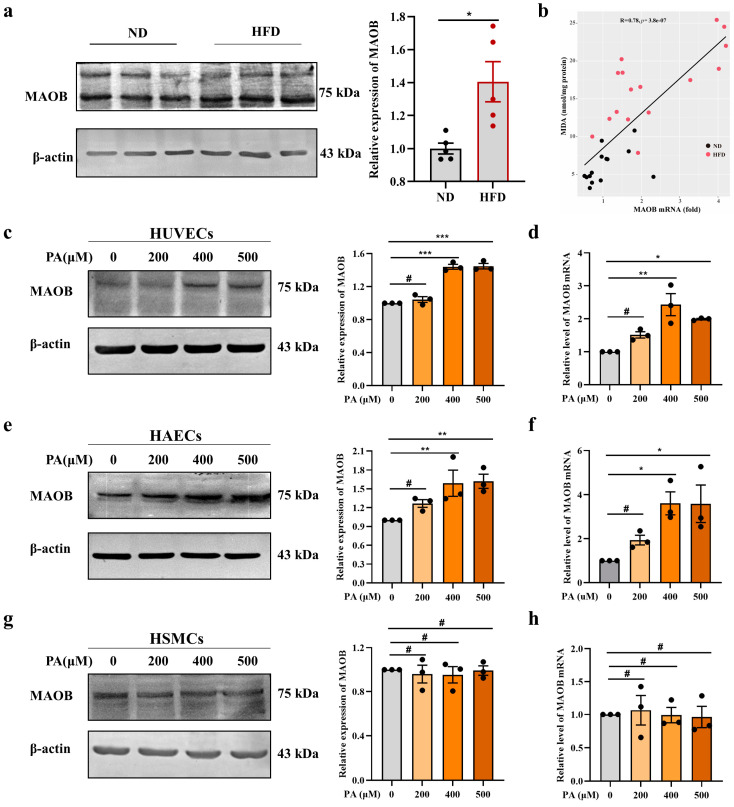
MAOB expression was increased in HFD-exposed vascular endothelial cells. (**a**) The protein levels of MAOB in the aortas of HFD and ND mice were measured by Western blot assay, with quantitative data in the right, *n* = 5. (**b**) Spearman correlation analysis were used to characterize the relationship between MDA levels and MAOB expressions in mice aortic tissues (*n* = 30). (**c**) The protein and (**d**) mRNA expressions of MAOB in HUVECs treated with the indicated dosage of palmitic acid (PA) for 24 h. (**e**) The protein and (**f**) mRNA expressions of MAOB in HAECs treated with the indicated dosage of PA for 24 h. (**g**) The protein and (**h**) mRNA expressions of MAOB in HSMCs treated with the indicated dosage of PA for 24 h. Data shown are individual values with means ± SEM. Data were analyzed using unpaired two-tailed Student *t*-test (**a**) or a one-way ANOVA (**c**–**h**). ^#^ *p* > 0.05, * *p* < 0.05, ** *p* < 0.01, *** *p* < 0.001.

To determine the role of MAOB in an HFD-induced oxidative stress and endothelial dysfunction, we first examined MAOB expression in mice. The data showed that the levels of MAOB protein were significantly increased in the aortas of the HFD-fed mice compared to those in the ND mice (Figure 1a). Furthermore, the MAOB mRNA levels were positively correlated with MDA levels (Figure 1b). In vitro, MAOB protein and mRNA levels were significantly upregulated in a dose-dependent manner in the PA-exposed HUVECs (Figure 1c,d). Consistently, MAOB expression was increased in the PA-exposed HAECs (Figure 1e,f), whereas there was only a slight change in MAOB expression in the PA-exposed HSMCs (Figure 1g,h). The increased MAOB expression in vascular endothelial cells suggested an important role of endothelial MAOB in the regulation of the HFD-induced endothelial dysfunction.

### 3.2. MAOB Activation Mediated PA-Induced Endothelial Oxidative Stress and Dysfunction In Vitro

**Figure 2:**
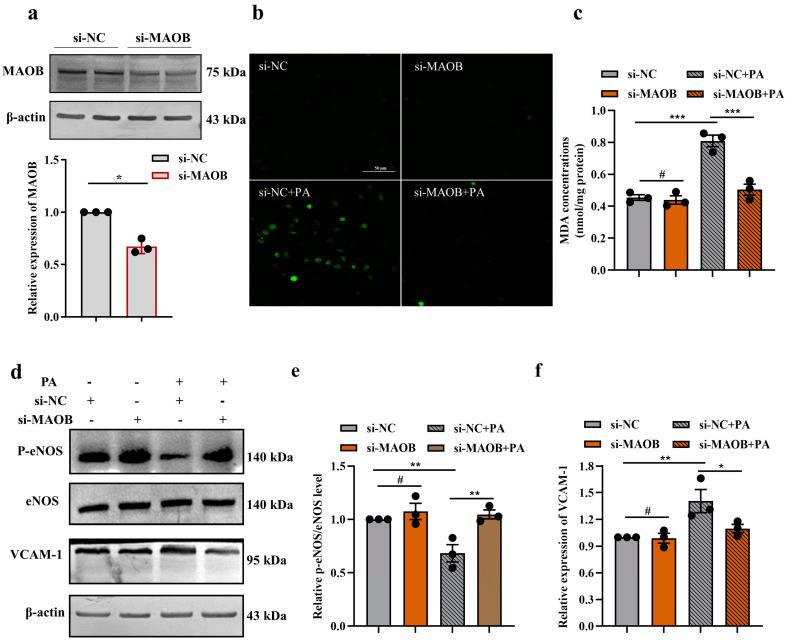
MAOB activation mediated PA−induced oxidative stress and endothelial dysfunction. HUVECs were transfected with MAOB siRNA, with or without PA (500 μmol/L) for 24 h. (**a**) MAOB protein expression was assayed by Western blot assay. (**b**) O_2_•^-^ production was stained by the fluorescent probe DCFH-DA (scale bar represented 50 µm). (**c**) MDA concentrations were determined using kits. (**d**) The p-eNOS and VCAM-1 protein levels were measured by Western blot assay and quantification of the blots (**e**,**f**). All values are presented as means ± SEM. Data were analyzed using unpaired two-tailed Student *t*-test (**a**) or a one-way ANOVA (**c**,**e**,**f**). ^#^ *p* > 0.05, * *p* < 0.05, ** *p* < 0.01, *** *p* < 0.001.

**Figure 3:**
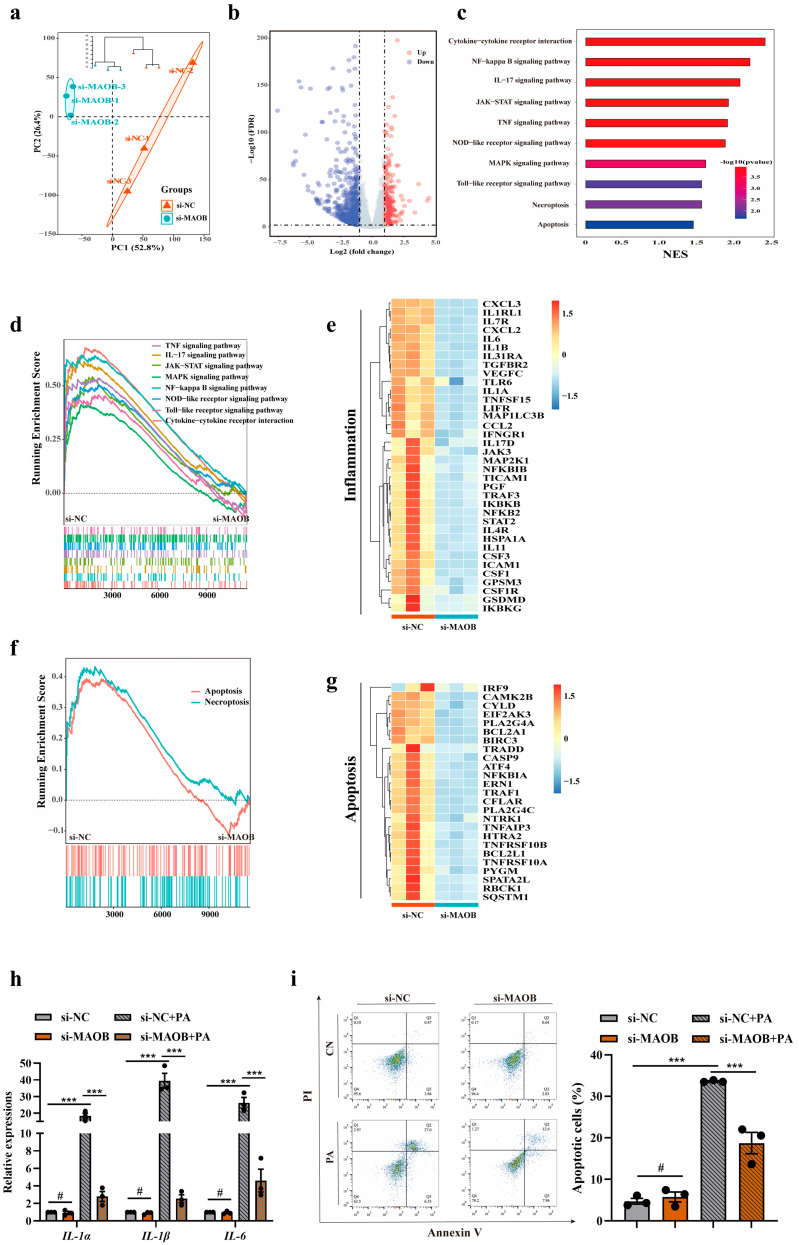
MAOB knockdown protected HUVECs against PA−induced inflammation response and cell apoptosis. RNA-sequencing (RNA-seq) analysis revealed key differential pathways and targets between the si-MAOB and si-NC transfected HUVECs under PA exposure. (**a**) PCA and unsupervised hierarchical clustering analysis of the RNA-seq data; *n* = 3. (**b**) Volcano plots for all differentially expressed genes. GSEA of pathways related to inflammation and apoptosis (**c**,**d**,**f**), and heatmaps of gene expression profiles related to inflammation (**e**) and apoptosis (**g**) based on the RNA-seq data set. Relative mRNA levels of inflammatory response genes (IL-1β, IL-1α and IL-6) (**h**) were detected, and cell apoptosis (**i**) was determined using Annexin V-FITC/PI apoptosis detection kit, and the percentages of positive apoptotic cells were detected by flow cytometry in HUVECs transfected with MAOB siRNA, with or without PA (500 μmol/L) for 24 h. Values are presented as means ± SEM. Data were analyzed by one-way ANOVA (**h**,**i**). ^#^ *p* > 0.05, *** *p* < 0.001.

To determine whether MAOB activation plays an essential role in oxidative stress and endothelial dysfunction in PA-treated HUVECs, we first silenced the MAOB gene by transfecting MAOB siRNA into HUVECs. Transfection efficacy was determined by Western blot assay. MAOB protein levels were decreased by approximately 40% after transfection with MAOB siRNA (Figure 2a). We found that silencing of the MAOB expression significantly reduced the production of O_2_•^-^ and the concentration of the MDA induced by PA in HUVECs (Figure 2b,c). Furthermore, the levels of p-eNOS/eNOS in the PA-treated HUVECs were dramatically elevated, and the VCAM-1 levels were reduced by transfecting MAOB siRNA (Figure 2d–f). To clarify the precise molecular pathway regulated by MAOB in endothelial cells under high-fat condition in an unbiased manner, we performed RNA-seq on PA-treated HUVECs with MAOB knockdown. Unsupervised PCA and hierarchical clustering clearly separated the samples into two clusters (Figure 3a). There were 1066 genes (315 upregulated and 751 downregulated) differentially expressed by the MAOB siRNA transfection in the PA-treated HUVECs (Figure 3b). GSEA revealed that cellular signaling pathways related to inflammation (such as NF-kappa B signaling pathway and TNF signaling pathway) and cell apoptosis (necroptosis and apoptosis) were enriched (Figure 3c) and significantly downregulated by MAOB knockdown (Figure 3d,f). Furthermore, a heatmap based on GSEA revealed that the expression of genes related to inflammation and the apoptosis pathway were significantly downregulated by the MAOB knockdown in the PA-treated HUVECs (Figure 3e,g). We also validated the transcriptome data in HUVECs, and found that the inflammatory response gene expression and apoptosis ratio were markedly inhibited by the MAOB knockdown under PA exposure (Figure 3h,i). Collectively, these results indicated that MAOB activation mediated endothelial oxidative stress and dysfunction induced by PA.

### 3.3. MAOB Expression Was Regulated by miR-3620-5p in HFD-Exposed Vascular Endothelial Cells

**Figure 4:**
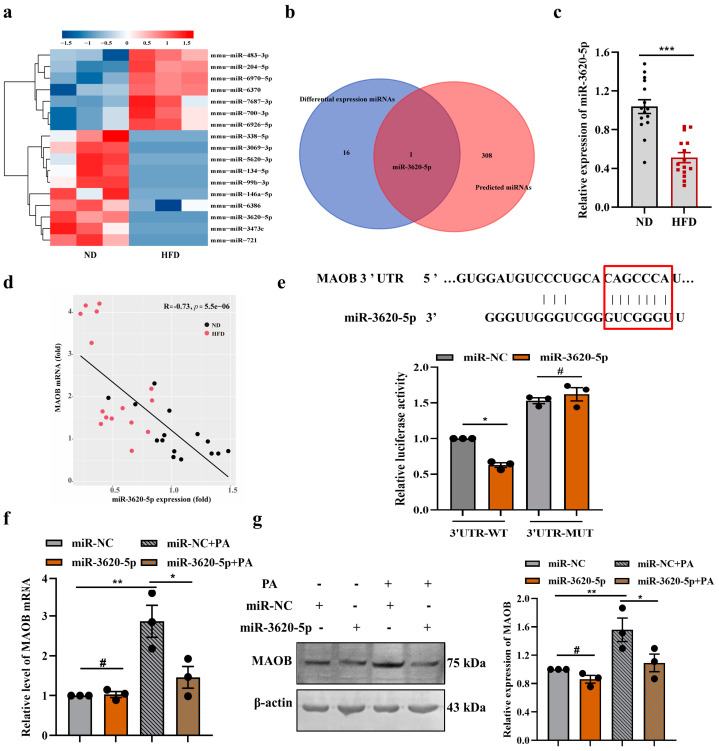
miR-3620-5p was an upstream regulator of elevated MAOB. (**a**) Heatmap of differentially expressed miRNAs in aortas from C57BL/6 mice fed an HFD for 16 weeks compared with that of ND mice by miRNA microarray analysis (*n* = 3). (**b**) Venn diagram showing the intersection of the miRNA identified by the microarray analysis and predicted by TargetScan database. (**c**) miR-3620-5p expressions were assayed by qPCR in the aortas of HFD or ND mice. (**d**) Spearman correlation analysis were used to characterize the relationship between MAOB mRNA levels and miR-3620-5p expressions in mice aortic tissues (*n* = 30). (**e**) Schematic representation of miR-3620-5p putative target sites in MAOB 3′-UTR, and luciferase reporter activities from HEK293 cells co-transfected with miR-3620-5p mimics or negative control and 3′ UTR of MAOB mRNA plasmid. (**f**) The mRNA and (**g**) protein expressions of MAOB in HUVECs transfected with miR-3620-5p mimics (50 nmol/L) with or without PA (500 μmol/L) treatment for 24 h. Data shown are individual values with means ± SEM. Data were analyzed using unpaired two-tailed Student *t*-test (**c**) or a one-way ANOVA (**e**–**g**). ^#^ *p* > 0.05, * *p* < 0.05, ** *p* < 0.01, *** *p* < 0.001.

Given that miRNAs act as novel regulators of MAOB at the post-transcriptional level [20], we hypothesized that MAOB gene expressions might be governed by posttranscriptional mechanisms such as the miRNA-induced inhibition of translation. To identify miRNAs potentially involved in an HFD-induced endothelial dysfunction, we examined the miRNA expression profiles in the aortas of HFD and ND mice (*n* = 3 per group). Heatmap analysis revealed that 17 miRNAs were identified as significantly up- or down-regulated compared with their levels in the ND group (Figure 4a). In silico prediction of miRNA-target gene interaction using the TargetScan database revealed possible interaction between miR-3620-5p and MAOB (Figure 4b). The expressions of miR-3620-5p were detected by qPCR in the HFD-mice aortas, appearing downregulated nearly 57% (Figure 4c). Next, we tested if an increase in MAOB was related to the miR-3620-5p expression suppression in mice aortas. The outcome affirmed the negative correlation between the miR-3620-5p and MAOB mRNA levels (Figure 4d). Then, we conducted luciferase reporter, qPCR and Western blot assays to experimentally clarify whether MAOB is a direct target for miR-3620-5p. We structured the pGLO-MAOB-3′UTR luciferase reporter plasmid and performed luciferase reporter assay in HEK293 cells. Our results demonstrated that co-transfection of miR-3620-5p with the MAOB 3′UTR reporter plasmid resulted in a significant inhibition of luciferase activity, whereas mutation of the binding sites abolished the suppressant effect of miR-3620-5p on the MAOB gene (Figure 4e). As shown in Figure 4f, transfection with miR-3620-5p mimics (50 nmol/L) potently decreased the MAOB mRNA expression compared with negative control cells. Moreover, similar results were observed at protein levels (Figure 4g). In summary, these results revealed that MAOB is a direct target of miR-3620-5p.

### 3.4. Selegiline Prevented Atherosclerosis by Attenuating Endothelial Oxidative Stress and Dysfunction

**Figure 5:**
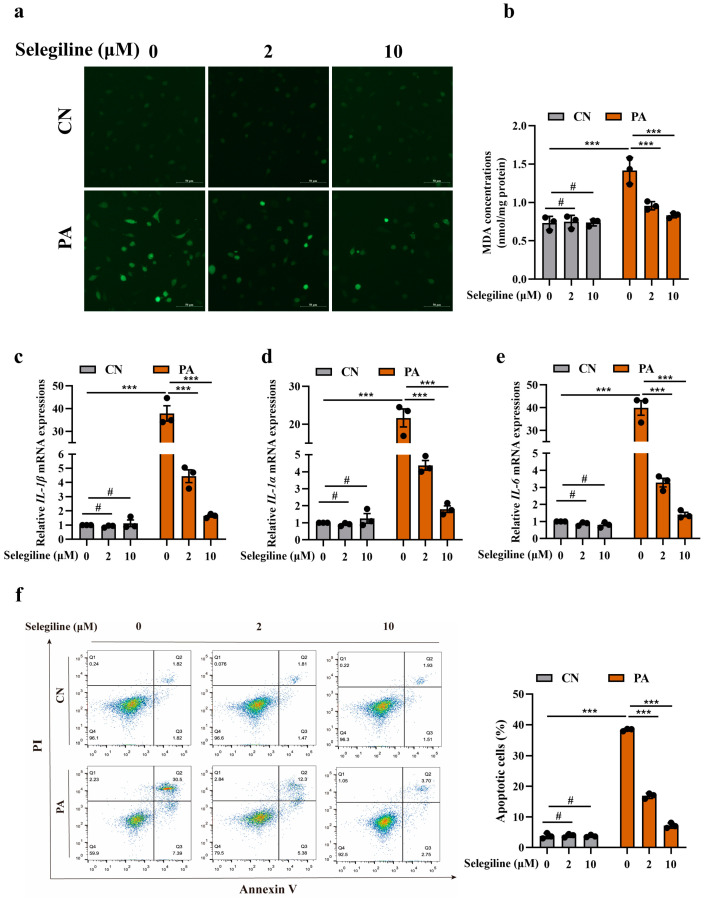
Selegiline reduced oxidative stress, inflammation, and apoptosis in HUVECs treated with PA. (**a**) Representative ROS staining images of HUVECs stimulated with PA (500 μmol/L) and treated with selegiline (2 and 10 μmol/L) for 24 h (scale bar represented 50 µm). (**b**) MDA concentrations were determined using kits. (**c**–**e**) The mRNA levels of genes related to inflammation (IL-1β, IL-1α and IL-6) in HUVECs in the indicated groups were assayed by qPCR. (**f**) Cell apoptosis was determined using Annexin V-FITC/PI apoptosis detection kit, and the percentages of positive apoptotic cells were detected by flow cytometry. All values are presented as means ± SEM. Data were analyzed using a one-way ANOVA. ^#^ *p* > 0.05, *** *p* < 0.001.

**Figure 6:**
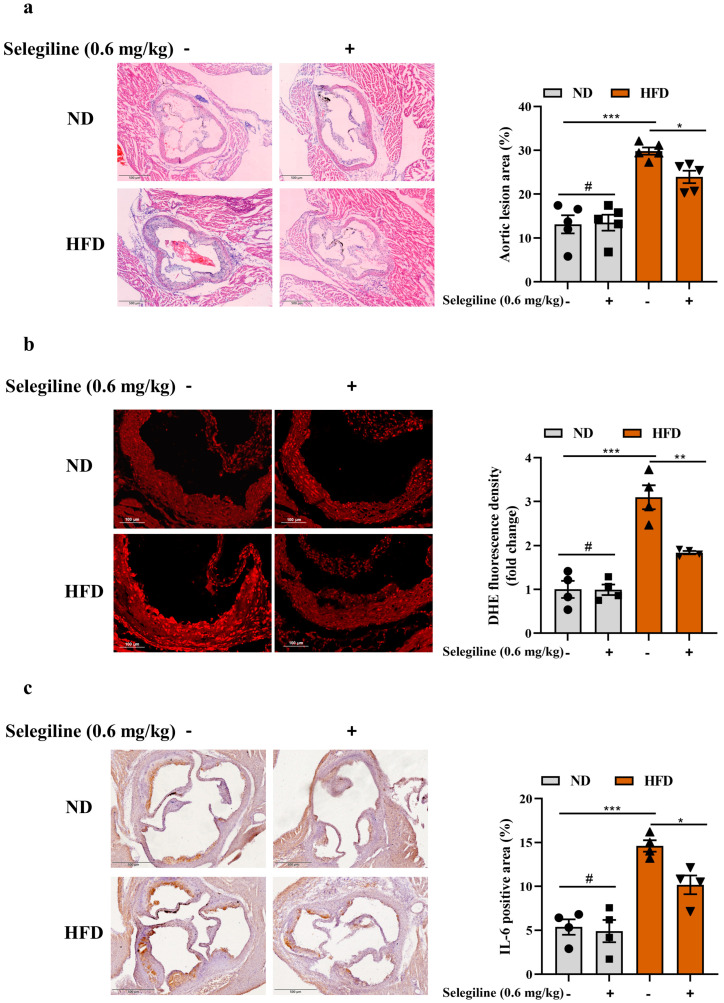
Selegiline attenuated atherosclerotic burden in *ApoE^−/−^* mice fed with HFD. (**a**) The representative images of H&E staining of atherosclerotic lesions of aortic root in *ApoE^−/−^* mice in the indicated groups, with quantitative data on the right; *n* = 5. DHE staining (**b**) and IL-6 immunostaining (**c**) of aortic root from ND or HFD-fed *ApoE^−/−^* mice with or without selegiline administration, with quantitative data on the right; *n* = 4. All values are presented as means ± SEM. Data were analyzed using a one-way ANOVA. ^#^ *p* > 0.05, * *p* < 0.05, ** *p* < 0.01, *** *p* < 0.001.

Due to the crucial role of MAOB activation in the progression of endothelial oxidative stress and dysfunction, it is worthwhile investigating whether MAOB inhibitors can exert beneficial therapeutic effects on atherosclerosis. Thus, we choose selegiline, an agent that can inhibit MAOB, to determine whether it can alleviate endothelial oxidative stress and dysfunction. To investigate the effects of selegiline on endothelial oxidative stress and dysfunction under high-fat stress, HUVECs were treated with selegiline (2 and 10 μmol/L) in the presence or absence of PA (500 μmol/L) stimulation for 24 h. ROS staining showed that cellular O_2_•^-^ accumulation was notably decreased by selegiline (Figure 5a). The antioxidative effect of selegiline was confirmed by the reduction of cellular MDA levels (Figure 5b). Furthermore, selegiline significantly attenuated PA-induced up-regulation of inflammatory genes (IL-1β, IL-1α and IL-6) (Figure 5c–e) and the apoptosis ratio (Figure 5f) in HUVECs. Given the antioxidative effects of selegiline in in vitro assays, we next examined whether this compound attenuated oxidative stress and atherosclerotic burden in *ApoE^−/−^* mice fed with HFD. *ApoE^−/−^* mice were fed with HFD for 10 weeks and then administered selegiline (0.6 mg/kg) by intraperitoneal injection for an additional 6 weeks with continuous HFD feeding. Selegiline treatment significantly reduced serum TC, TG, LDL-C, glucose, ALT, and AST levels in HFD fed *ApoE^−/−^* mice (Supplementary Figure S3). Notably, compared to those of the control HFD *ApoE^−/−^* mice, a significant decrease in plaque area was observed in the HFD fed *ApoE^−/−^* mice with selegiline administration (Figure 6a). Accordingly, selegiline treatment effectively reversed the elevated production of O_2_•^-^ and the level of inflammatory cytokine IL-6 in HFD fed *ApoE^−/−^* mice (Figure 6b,c). Taken together, our data indicated that the administration of selegiline significantly ameliorated endothelial dysfunction and reduced atherosclerotic burden in the HFD-fed *ApoE^−/−^* mice.

### 3.5. Selegiline Administration Altered the Composition of Gut Microbiota in ApoE^−/−^ Mice

**Figure 7:**
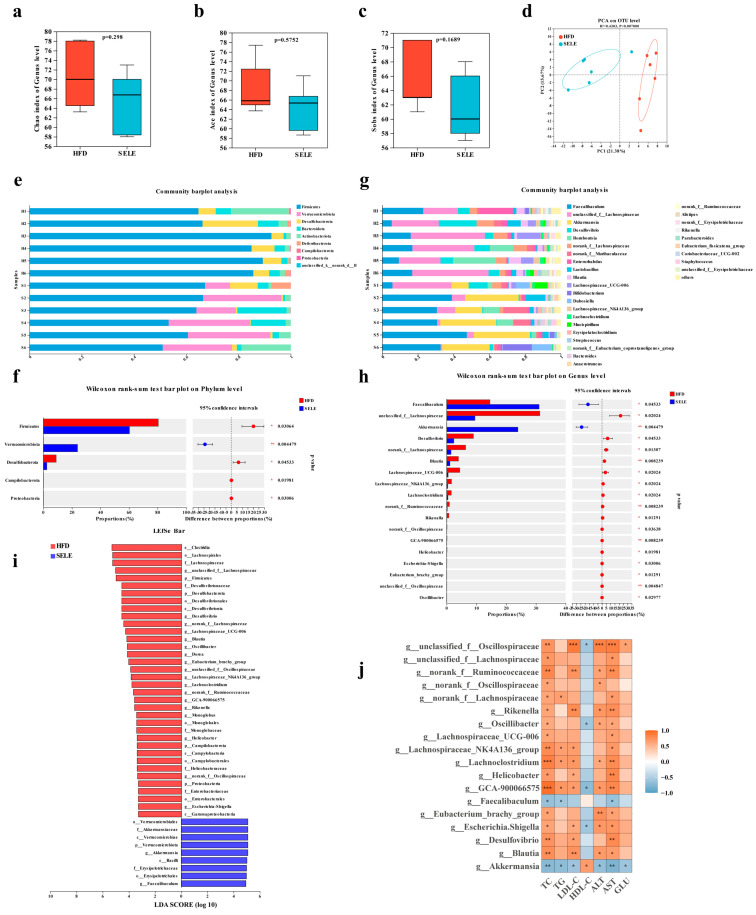
Selegiline significantly altered the composition of gut microbiota in HFD fed *ApoE*^−/−^ mice. Feces of each mouse were collected after 6 weeks of treatment with or without selegiline, and analyzed by utilization of 16S rRNA sequencing. (**a**–**c**) Diversity of the gut microbiota assessed by Chao, Ace, and Sobs indices among the HFD and selegiline treatment group. (**d**) Principal component analysis (PCA) score plot. (**e**,**f**) Taxonomic composition and Wilcoxon rank-sum test at the phylum level. (**g**,**h**) Taxonomic composition and Wilcoxon rank-sum test at the genus level. (**i**) Different gut microbiota taxa enriched in the HFD and selegiline group by LEfSe. (**j**) Correlation between significant gut microbiota (the relative abundances of the 18 different genera) and serum biochemical parameters by Spearman correlation analysis; red indicates a positive correlation and blue indicates a negative correlation. *n* = 6 in each group; * *p* < 0.05, ** *p* < 0.01, *** *p* < 0.001.

The gut microbiota is associated with cardiovascular diseases, including the progression of atherosclerosis. The influence of selegiline on gut microbiota under atherosclerotic condition remains unknown. Hence, we analyzed the gut microbiota composition in HFD-fed *ApoE^−/−^* mice with or without selegiline treatment by 16S rRNA sequencing. Microbial richness and diversity (Chao, Ace, Sobs, Shannon and Pd indices) tended to be lower than in the HFD group after selegiline treatment, but this was not significant (Figure 7a–c and Figure S4a,b). PCA and PCoA score plots showed marked separation between the HFD group and the selegiline group (Figure 7d and Figure S4c), indicating that selegiline administration could significantly alter the composition of the gut microbiota. Next, we investigated the differences in gut microbiota at different taxonomic levels. At the phylum level, the relative abundances of Firmicutes and Desulfobacterota were decreased after selegiline administration, and that of Verrucomicrobiota was significantly increased compared with that of the HFD-fed mice (Figure 7e,f). Furthermore, selegiline treatment showed a modestly, albeit not significantly, decreased Firmicutes/Bacteroidota ratio in the HFD-fed mice, suggesting a potential beneficial effect of selegiline on the gut microbiota composition (Supplementary Figure S4d). At the genus level, unclassified_f__Lachnospiraceae was the predominant species in the HFD group, while *Faecalibaculum* and *Akkermansia* constituted the majority in the selegiline group (Figure 7g). Two genera, *Faecalibaculum* and *Akkermansia*, were enriched in the selegiline group, while sixteen other genera, including unclassified_f__Lachnospiraceae, Desulfovibrio, and Blautia, were depleted compared with the HFD group (Figure 7h). The taxonomic cladograms from the LEfSe analysis confirmed that the changed gut microbiome could be screened as the biological markers to distinguish the selegiline and the HFD group (Figure 7i and Figure S4e).

As selegiline significantly altered the composition of the gut microbiota and improved serum biochemical levels in the HFD-fed *ApoE^−/−^* mice, we further analyzed the correlation between different gut microbiota and serum biochemical parameters using the Spearman correlation. Interestingly, selegiline-enriched *Faecalibaculum* and *Akkermansia* were negatively related with TC, TG, LDL-C, ALT, AST, GLU, and positively related to HDL-C, while the selegiline-depleted genera including Blautia and Desulfovibrio were positively related to TC, TG, LDL-C, ALT, AST, GLU, and inversely related to HDL-C (Figure 7j). These findings indicated that the change in gut microbiota associated with selegiline intervention was potentially correlated with the improvement in lipid metabolism.

## 4. Discussion

Taken together, our results showed the essential role of MAOB in the regulation of endothelial oxidative stress homeostasis, and identified the miR-3620-5p as the upstream regulator of elevated MAOB. In addition, our findings also revealed that selegiline, a MAOB pharmacal inhibitor, exerted an anti-atherosclerotic effect not only through its protective effect against endothelial dysfunction, but also partly by modulating the composition and function of the gut microbiome, which provided evidence for selegiline as a potential drug for treating ACVDs.

Endothelial dysfunction is the first step in a complex and multifaceted process that eventually leads to atherosclerosis and ACVDs [5]. ROS in endothelial cells lies at the core of oxidative stress biology [21]. Endothelium-dependent factors, such as NO and NO-containing intermediates, are involved in the production of free radicals and play pivotal roles in endothelial dysfunction [22]. Decreased NO bioavailability may be related to NO reduction and enhanced reaction of NO with O_2_•^-^, resulting in increased formation of ONOO^−^ [1]. ROS executes a vital effect on the inflammatory response, cell apoptosis, and NF-κB signaling activation [23]. In response to the HFD exposure, endothelial cells in the lining of vessels undergo oxidative stress and secrete various cellular adhesion cytokines, such as VCAM-1, ICAM-1, and E-selectin. In line with a previous study, we detected oxidative stress, eNOS activity and adhesion cytokine and apoptosis of endothelial cells in the aortas of the HFD-fed mice (Supplementary Figure S1). Importantly, we found that MAOB expression was increased in the aortas of the HFD-fed mice. The previous studies reported that MAOB was expressed in the atherosclerotic plaques; however, the evidence of cell-specific MAOB involvement in atherogenesis is lacking. Here, in vitro, we found that MAOB expressions were increased in the PA-exposed HUVECs and HAECs, only slightly changed in the PA-exposed HSMCs, indicating the importance of MAOB in maintaining endothelial homeostasis that is essential to normal vascular physiology.

MAOs are redox enzymes which generate H_2_O_2_ as a by-product of their catalytic cycle [14]. MAOs are another source of ROS, and their relevance to vascular ROS formation has gained more attention. It has been shown that inhibition of MAOB restored endothelial dysfunction and reduced atherosclerosis by inhibiting several key steps (ROS, adhesion molecules and proinflammatory cytokines) in its pathogenesis [14,16]. However, an important limitation of a previous study is that a significant part of the authors’ observations was only based on pharmacological MAOB inhibition. In this study, we used MAOB siRNA to downregulate the expression of MAOB in HUVECs. We found that MAOB siRNA significantly attenuated cellular oxidative stress levels and alleviated endothelial dysfunction induced by PA treatment. Furthermore, using an unbiased RNA-seq analysis, we found that MAOB knockdown largely decreased the expression of genes associated with inflammation and apoptosis in HUVECs treated with PA. These findings revealed the important role of MAOB in endothelial oxidative stress and dysfunction.

Accruing evidence has highlighted the importance of miRNAs in all stages of atherosclerosis via the post-transcription regulation of atherosclerosis-prone genes [24,25]. We conducted a microarray analysis to detect miRNAs differentially expressed between HFD and ND fed mice aortas, and identified significantly decreased miR-3620-5p in HFD fed mice, as further verified in HUVECs treated with PA (Supplementary Figure S2a). Interestingly, bioinformatics analysis predicted a complementary binding between miR-3620-5p and MAOB mRNA 3′UTR. Our results also confirmed that miR-3620-5p mimic inhibited MAOB expression by directly binding to its 3′UTR. In several studies, miR-3620-5p was found upregulated in intramucosal gastric cancer and glioma related to cellular proliferation [26,27]. To the best of our knowledge, no previous research has reported the functional role of miR-3620-5p in vascular endothelial cells; however, in vivo evidence of miR-3620-5p involvement in endothelial function and atherogenesis is lacking; further studies need to explore the miR-3620-5p role in endothelial function using cell-specific miR-3620-5p knockout mice.

The conventional treatment modalities for ACVDs focus on lipid modulation and platelet aggregation inhibition. Despite significant advances in the clinical treatment of ACVDs, this problem is still largely unsolved, and alternative therapeutic strategies need to be investigated. Due to the crucial role of MAOB in endothelial dysfunction and atherosclerosis, it is worthwhile investigating whether MAOB inhibitors can exert beneficial effects on ACVDs. Selegiline (previously called L-deprenyl) is a clinically widely used, irreversible and selective inhibitor of MAOB, which is primarily used to treat Parkinson’s disease and depression [28,29]. We and others have reported that selegiline significantly decreased fatty liver in rodent animals fed with a lipid-rich diet [16,17,30]. Only one recent study revealed that selegiline reduced atherosclerosis by inhibiting ROS production, plasma LDL-C levels, the expression of adhesion molecules and proinflammatory cytokines [16]. Consistent with these results, we observed that selegiline treatment could significantly alleviate oxidative stress, inflammation, and apoptosis in PA-treated HUVECs, as well as decrease ROS production, inflammatory cytokine, and plaque formation in HFD-fed *ApoE^−/−^* mice. Combined with the previous reports, our findings support the view that selegiline reduces atherosclerosis likely through its multiple protective effect against endothelial dysfunction.

The gut microbiota was found to be strongly associated with atherosclerosis [31,32]. Evidence from numerous studies showed that dysbiosis of gut microbiota could promote the development of atherosclerosis [33,34], and modulation of gut microbiota was considered a potential therapeutic target of atherosclerosis [35,36,37,38]. To explore the relationship between the anti-atherosclerosis effect of selegiline and gut microbiota, 16S rRNA sequencing was used to observe the changes in gut microbiota in HFD-fed *ApoE^−/−^* mice treated with selegiline. Alpha diversity analysis did not reveal a significant decrease in fecal microbial diversity after selegiline treatment. A series of studies on gut microbiota revealed the increased Firmicutes/Bacteroidetes ratio in *ApoE^−/−^* mice fed a Western diet as well as patients with ACVDs [39,40]. In this study, we found that selegiline treatment modestly reduced the Firmicutes/Bacteroidetes ratio, which implies that selegiline exhibits a beneficial effect on HFD-associated gut microbiota dysbiosis. Compared to the HFD group, *Faecalibaculum* and *Akkermansia* genera were significantly increased after selegiline treatment along with a negative correlation to serum biochemical levels. The abundance of genus *Faecalibaculum* was reported to be strongly positively correlated with short-chain fatty acid (SCFA) production [41,42]. In the gut, SCFAs are one of the most important microbial metabolites, and their beneficial role in regulating inflammation and metabolism has been reported [43]. *Akkermansia* accounts for 1–3% of the healthy subject intestinal microbiota [44], which has beneficial effects on combating obesity-related metabolic disorders [45,46]. Moreover, a previous study reported that *Akkermansia* muciniphila treatment by oral gavage substantially reduced HFD-induced atherosclerosis by ameliorating both aortic and systemic metabolic inflammation [44]. The above evidence strongly implies that *Faecalibaculum* and *Akkermansia* might somehow exhibit a beneficial effect on the improvement of atherosclerosis associated with selegiline. Furthermore, we found that unclassified_f__Lachnospiraceae, Desulfovibrio, and Blautia genera were depleted after selegiline treatment; they were positively associated with serum biochemical parameters. In line with our results, the abundance of unclassified_f__Lachnospiraceae has been reported to be positively connected with obesity-related indicators [47,48]. Desulfovibrio is also known as sulfate-reducing bacteria which can damage the gut barrier function by resulting in higher levels of circulating lipopolysaccharide [49]. Furthermore, Desulfovibrio was expected to be a potential biomarker and a therapeutic target for atherosclerosis [50]. It should be noted that the genus Blautia, as a member of the Lachnospiraceae family, could produce SCFAs which can regulate inflammation and metabolism [51]. However, in our research, we observed the decreased abundance of Blautia after selegiline treatment, and Spearman correlation analysis showed that lipid parameters were positively associated with Blautia, which suggests that Blautia may not be involved in the regulation of selegiline of lipid metabolism. Taken together, our data suggest a probable association between the amelioration of atherosclerosis and the changes in gut microbiota after selegiline administration.

In conclusion, we discovered that endothelial MAOB was activated under HFD exposure, which controlled endothelial oxidative stress homeostasis and atherogenesis. We also identified miR-3620-5p as the upstream regulator of elevated MAOB. In addition, the present study provided novel insights into the protective effect of selegiline on atherosclerosis, providing evidence for selegiline as a promising therapeutic strategy for ACVDs.

## Figures and Tables

**Table 1:** Primers used for qPCR analysis.

Gene	Primer Sequence (5′-3′)
has-miR-3620-5p	F: GTGGGCTGGGCTGGGC R: AGTGCAGGGTCCGAGGTATT
	R: AGTGCAGGGTCCGAGGTATT
mmu-miR-3620-5p	F: AACAATCTGTGGGCTGGGCTG
	R: ATCCAGTGCAGGGTCCGAGG
has-MAOB	F: GAAGAGTGGGACAACATGAC
	R: CTCCACACTGCTTCACATAC
mmu-MAOB	F: CAACAACCAATGGAGGACAGGAGAG
	R: CTGAACCCAAAGGCACACGAGAG
β-actin	F: ACTATCGGCAATGAGCG
	R: GAGCCAGGGCAGTAATCT
has-IL-6	F: GCCTTCGGTCCAGTTGCCTTC
	R: GCCTCTTTGCTGCTTTCACACATG
has-IL-1α	F: GACCAACCAGTGCTGCTGAAGG
	R: CTTAGTGCCGTGAGTTTCCCAGAAG
has-IL-1β	F: GGACAGGATATGGAGCAACAAGTGG
	R: CAACACGCAGGACAGGTACAGATTC

Note: The primers were obtained by Sangon Biotech (Shanghai, China).

## Data Availability

All data are available from the corresponding author upon reasonable request.

## Supplementary Materials

The following supporting information can be downloaded at: https://www.mdpi.com/article/10.3390/nu15112542/s1, Figure S1. Comparison of serum biochemical indices and vascular injury between the high fat diet (HFD) or normal diet (ND) fed mice for 16 weeks; Figure S2. The expressions of miR-3620-5p were assayed by qPCR in HUVECs; Figure S3. Selegiline ameliorated serum biochemical parameters in HFD fed *ApoE^−/−^* mice; Figure S4. Selegiline significantly altered the composition of gut microbiota in HFD fed *ApoE^−/−^* mice; Uncut Gel Blots.
