# Supplementary material for: Inhibition of MAOB Ameliorated High-Fat-Diet-Induced Atherosclerosis by Inhibiting Endothelial Dysfunction and Modulating Gut Microbiota
Source: Nutrients. 2023 May 30;15(11):2542. doi: 10.3390/nu15112542 (PMC10255117; doi:10.3390/nu15112542)
Supplement: Supplementary file 1 [file nutrients-15-02542-s001.zip › nutrients-2413295-supplementary.pdf]

Supplementary materials

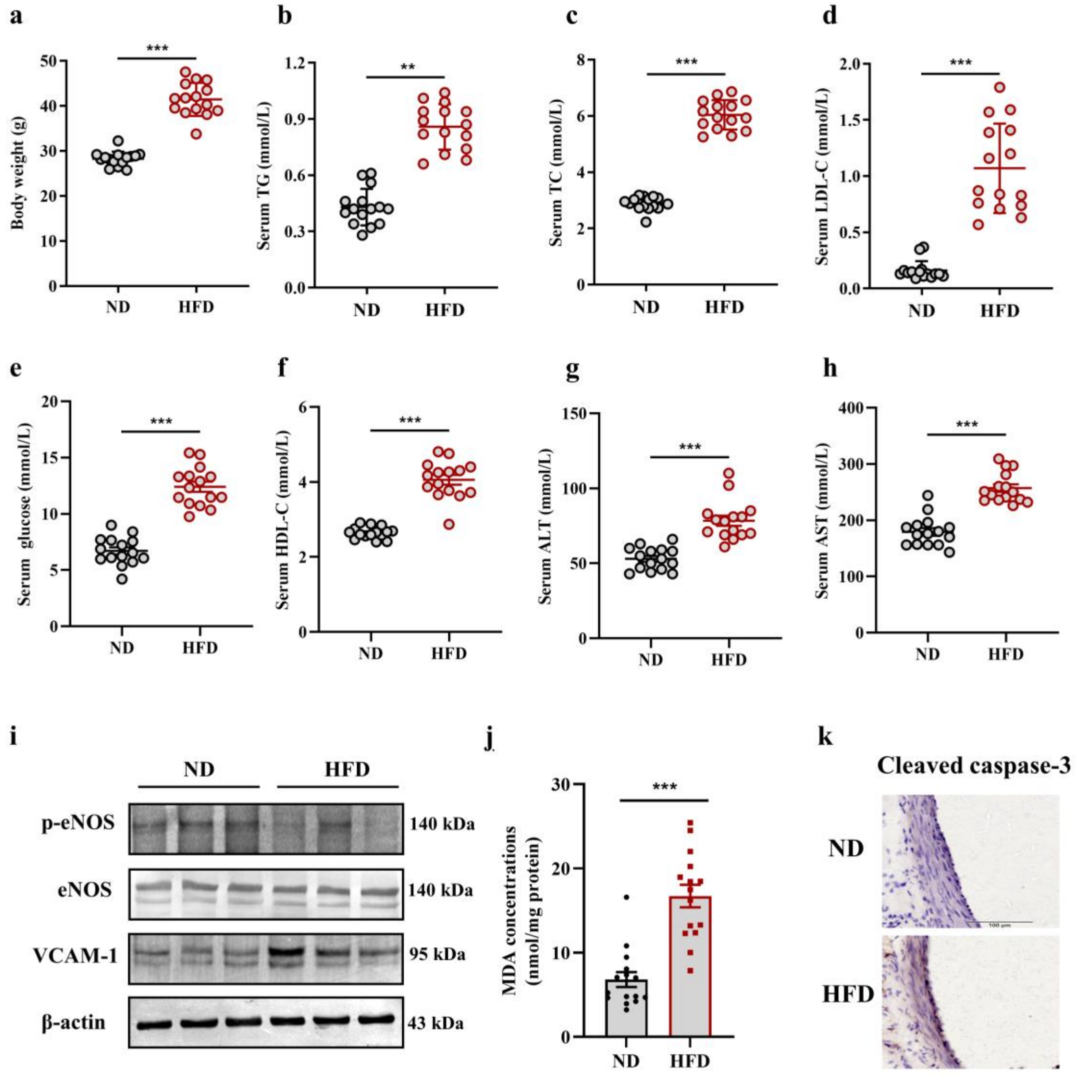

Supplementary Fig. 1 Comparison of serum biochemical indices and vascular injury between the high fat diet (HFD) or normal diet (ND) fed mice for 16 weeks. **(a)** Body weight, **(b)** serum triglyceride (TG), **(c)** serum total cholesterol (TC), **(d)** serum low density lipoprotein cholesterol (LDL-C), **(e)** serum glucose, **(f)** serum high density lipoprotein cholesterol (HDL-C), **(g)** serum alanine transaminase (ALT), **(h)** serum aspartate transaminase (AST). n=15 per group. **(i)** The endothelial dysfunction-

associated proteins p-eNOS and VCAM-1 expression in the aortas from HFD and ND fed mice were determined by western blot assay, n=3. **(j)** The MDA levels were determined by assay kits in HFD or ND fed mice aortas, n=15 in each group. **(k)** Cleaved caspase-3 immunostaining of aortic root from HFD and ND fed mice. Data shown were individual values with means ± SEM. Data were analyzed using unpaired two-tailed student t-test. $^{\#}p > 0.05$, $^{*}p < 0.05$, $^{**}p < 0.01$, $^{***}p < 0.001$.

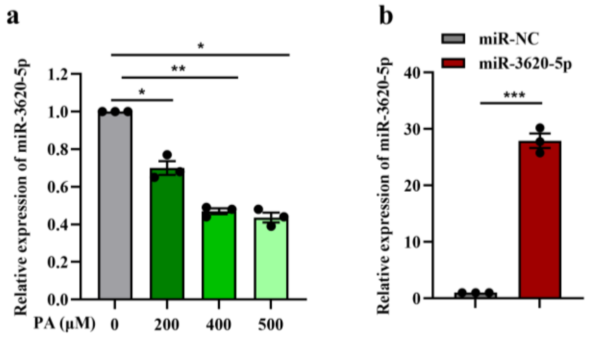

Supplementary Fig. 2 (a) The expressions of miR-3620-5p were assayed by qPCR in HUVECs treated with the indicated dosage of palmitic acid (PA) for 24 h. **(b)** Transfection efficiency was determined by qPCR in HUVECs were transfected miR-3620-5p mimics (50 nmol/L) or negative control (miR-NC) for 24 h. All values were presented as means ± SEM. Data were analyzed using a one-way ANOVA (a) or unpaired two-tailed student t-test (b). $^{\#}p > 0.05$, $^{*}p < 0.05$, $^{**}p < 0.01$, $^{***}p < 0.001$.

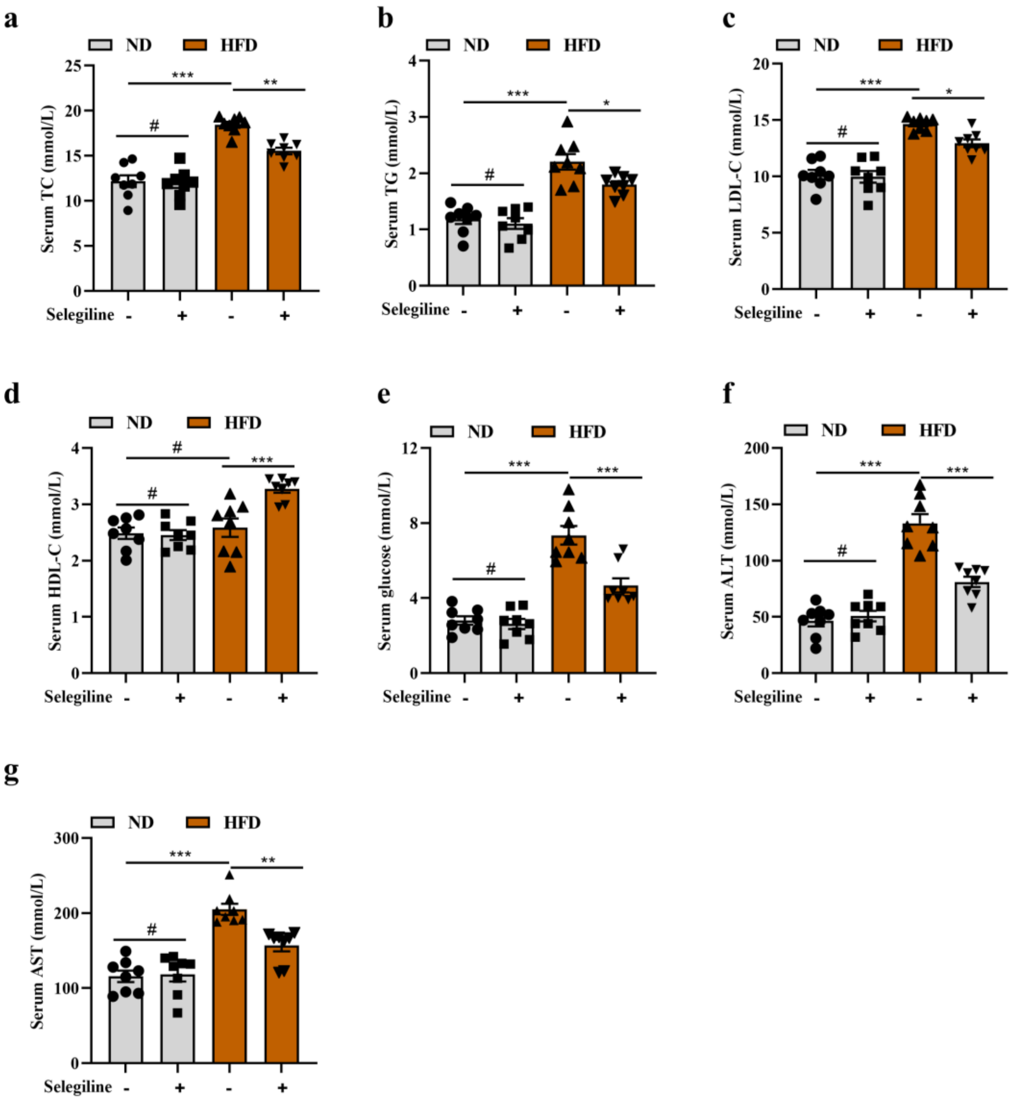

Supplementary Fig. 3 Selegiline ameliorated serum biochemical parameters in HFD fed *ApoE*$^{-/-}$ mice. (a) serum TC, **(b)** serum TG, **(c)** serum LDL-C, **(d)** Serum HDL-C, **(e)** serum glucose, **(f)** serum ALT, **(g)** serum AST. n=8 per group. All values were presented as means ± SEM. Data were analyzed using a one-way ANOVA. $^{\#}p > 0.05$, $^{*}p < 0.05$, $^{**}p < 0.01$, $^{***}p < 0.001$.

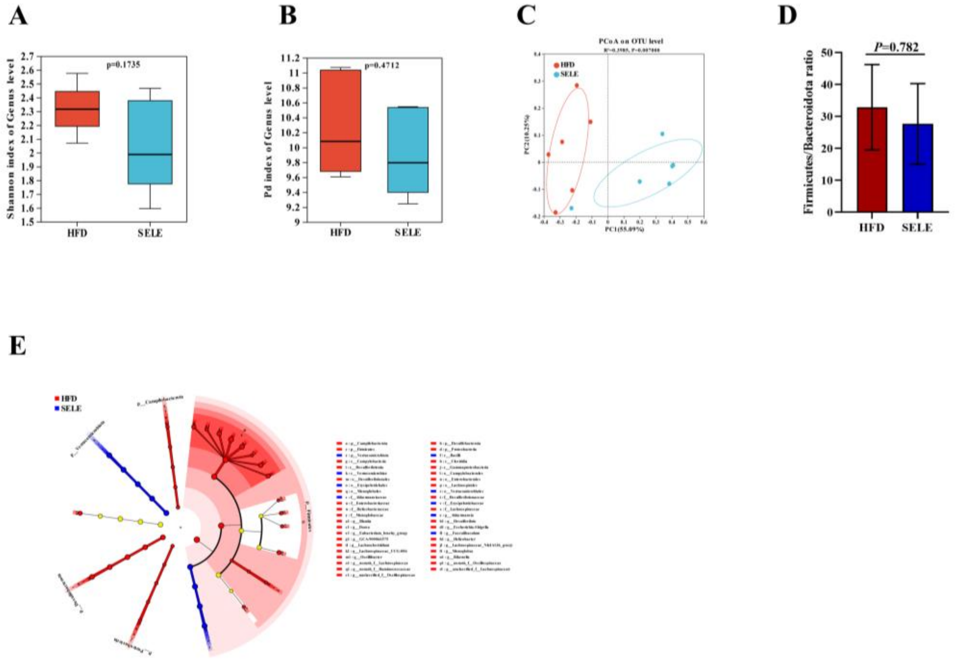

Supplementary Fig. 4 (a) Shannon, and **(b)** Pd index in alpha diversity analysis among the HFD and HFD with selegiline treatment group. **(c)** Principal Coordinate Analysis (PCoA) based on the Bray Curtis distance algorithm. **(d)** The Firmicutes/Bacteroidetes ratio between the two groups. **(e)** Taxonomic cladogram generated by LEfSe analysis showing taxa significantly enriched in HFD (red) or HFD with selegiline treatment (blue) group, respectively.
